# Structural studies of novel glycoconjugates from polymerized allergens (allergoids) and mannans as allergy vaccines

**DOI:** 10.1007/s10719-015-9640-4

**Published:** 2015-11-25

**Authors:** Ana I. Manzano, F. Javier Cañada, Bárbara Cases, Sofia Sirvent, Irene Soria, Oscar Palomares, Enrique Fernández-Caldas, Miguel Casanovas, Jesús Jiménez-Barbero, José L. Subiza

**Affiliations:** Centro de Investigaciones Biológicas, Madrid, Spain; Inmunotek SL, Punto Mobi 5, Alcalá de Henares, Madrid Spain; Facultad de Químicas, Universidad Complutense, Madrid, Spain

**Keywords:** Mannan, Allergen, Allergoid, Glutaraldehyde, Conjugation, Vaccine

## Abstract

Immunotherapy for treating IgE-mediated allergies requires high doses of the corresponding allergen. This may result in undesired side effects and, to avoid them, hypoallergenic allergens (allergoids) polymerized with glutaraldehyde are commonly used. Targeting allergoids to dendritic cells to enhance cell uptake may result in a more effective immunotherapy. Allergoids coupled to yeast mannan, as source of polymannoses, would be suitable for this purpose, since mannose-binding receptors are expressed on these cells. Conventional conjugation procedures of mannan to proteins use oxidized mannan to release reactive aldehydes able to bind to free amino groups in the protein; yet, allergoids lack these latter because their previous treatment with glutaraldehyde. The aim of this study was to obtain allergoids conjugated to mannan by an alternative approach based on just glutaraldehyde treatment, taking advantage of the mannoprotein bound to the polymannose backbone. Allergoid-mannan glycoconjugates were produced in a single step by treating with glutaraldehyde a defined mixture of allergens derived from *Phleum pratense* grass pollen and native mannan (non-oxidized) from *Saccharomyces cerevisae*. Analytical and structural studies, including 2D-DOSY and ^1^H-^13^C HSQC nuclear magnetic resonance spectra, demonstrated the feasibility of such an approach. The glycoconjugates obtained were polymers of high molecular weight showing a higher stability than the native allergen or the conventional allergoid without mannan. The allergoid-mannan glycoconjugates were hypoallergenic as detected by the IgE reactivity with sera from grass allergic patients, even with lower reactivity than conventional allergoid without mannan. Thus, stable hypoallergenic allergoids conjugated to mannan suitable for using in immunotherapy can be achieved using glutaraldehyde. In contrast to mannan oxidation, the glutaraldehyde approach allows to preserve mannoses with their native geometry, which may be functionally important for its receptor-mediated recognition.

## Introduction

The immunotherapy of IgE-mediated allergic diseases is based on the administration of increasing amounts of allergens to desensitize allergic patients (allergen vaccines). For immunotherapy to be effective, high doses of allergens have to be administered [1]. This raises major safety concerns due to the sensitivity of the patients to the allergens that the vaccine contains [1].

Immunotherapy with modified allergens to render them hypoallergenic (allergoids) is increasingly being used, since the safety profile of allergoids allows to a faster and simpler dosing than with native (non-modified) allergens [2, 3]. Glutaraldehyde is the most widely used agent for allergoid formation [4, 5]. By means of its two reactive aldehyde groups, it cross-links the allergen proteins through the ε-amino groups of lysine residues. This reaction results in allergen polymerization, with the concomitant loss of accessibility of IgE antibodies to the allergen epitopes, i.e., antibody binding sites, [4].

The goal of allergy vaccines is to induce a therapeutic immune response against the corresponding allergens. The main initiators of such a response are dendritic cells (DCs), which are continuously sampling antigens from the microenvironment by receptor-mediated endocytosis, micropinocytosis and phagocytosis [6]. C-type lectin receptors (CLRs) are pivotal for recognizing glycans, in a calcium-dependent manner [7]. Key examples are mannose receptor (MR), Dectin-2 and DC-SIGN, which preferentially recognize mannose residues [8]. Therefore, antigen conjugation with mannan, as a source of polymannoses, has been proposed to increase the antigen uptake by DCs [9], including allergens [10]. While this concept is of paramount interest for vaccine development, the notion is even more evident when considering hypoallergenic polymerized allergens, since it has been claimed that these polymers are not efficiently captured by DCs [11].

Conventional methodologies for coupling mannan to proteins require, as a first step, its prior oxidation (oxidized mannan) to release reactive aldehyde groups (−CHO) able to bind to the protein amino groups [12]. This approach is however not suitable for conjugating mannan to allergoids, because the dramatic reduction of free amino groups once the protein has reacted with glutaraldehyde [13].

Here we show a novel approach to obtain allergoid-mannan glycoconjugates. Our concept is based on using just a glutaraldehyde treatment, taking advantage of the mannoprotein bound to the polymannose backbone of mannan. Analytical and structural studies show the feasibility of such an approach, which results in a high molecular weight and stable structure with a reduced IgE binding reactivity suitable to be used for allergen immunotherapy.

## Material and methods

### Allergens

Defatted grass pollen grains from *Phleum pratense* (Iberpolen, Jaén, Spain) were extracted overnight with phosphate buffered saline, pH 7.2 (PBS) and submitted to tangential flow ultrafiltration (cut off pore size, 100 kDa). The enriched allergen fraction obtained in the filtrate was dialyzed with distilled water and lyophilized in small aliquots until used. Total protein content was measured by the Bradford assay using serum albumin as standard (Bio-Rad Laboratories, Madrid, Spain).

### Mannan from *S. cerevisae*

Mannan was obtained as described [14, 15] with slight modifications. Briefly, mannan was extracted from yeast (*Saccharomyces cerevisae*; Lesaffre Ibérica, Madrid, Spain) in hot citrate buffer (0.02 M; pH 7.0) during 90 min. The extract was precipitated with ethanol and dialyzed against distilled water. Mannan was precipitated in the presence of cetavlon (Sigma-Aldrich, Madrid, Spain) (50 % *v*/*v*) after several hours in a shaker by adding 2 % borate sodium (pH 8.8). The precipitate was collected by centrifugation and washed twice with 2 % acetic acid in ethanol plus a final wash with 100 % ethanol. Once re-dissolved and dialyzed in distilled water, it was applied to a DEAE-Sephadex A-50 column equilibrated with 0.02 M Tris-HCl buffer (pH 7.5). A linear gradient from 0 to 0.5 M NaCl was used. Mannan-containing fractions (M) were pooled, dialyzed extensively against distilled water and lyophilized in aliquots until used.

### Allergen-mannan conjugation

Allergens from grass pollen *Phleum pratense* were polymerized and conjugated with mannan in a single step as follows. Glutaraldehyde (25 %, Sigma) was added to a solution (final concentration 25 mM) containing a mixture of the allergen and mannan in PBS. This mixture was made at different allergen:mannan ratios (1:4; 1:1; 1:0.5; 1:0.3; 1:0.15), using a fix protein amount and different amounts of freeze-dried mannan. Reaction was performed during 6 h at 4 °C in continuous stirring and stopped with glycine (1.25 M), followed by tangential flow filtration with distilled water (membrane cut off, 100 kDa) to remove free allergen and mannan molecules, virtually all of them below that size. Allergen-mannan (AM) conjugates were recovered in the concentrated retentate (>100 kDa fraction) that was further lyophilized until use. Total carbohydrates and protein content of reconstituted samples were measured by the anthrone [16] and Bradford [17] assays, respectively.

For control purposes, one part of the same allergen extract remained untreated (native allergen, N) or subjected to the above protocol but without mannan to obtain a conventional mannan-free polymerized allergen (POL).

### Gel electrophoresis (SDS-PAGE) and immunoblotting

Every allergen sample (N, POL and AM) was submitted to protein separation in 12.5 % polyacrylamide gels under denaturalizing conditions with sodium dodecyl sulfate and Coomassie blue staining. Immunoblots were performed transferring the proteins separated by electrophoresis to cellulose nitrate membranes (Bio Rad, Germany). The membranes were blocked with 5 % bovine serum albumin in PBS-0.1 % Tween 20 and incubated with a pooled serum from grass allergic patients. Afterwards, they were incubated with an anti-human IgE monoclonal antibody conjugated with peroxidase (Southern Biotech, USA) at a 1/2000 dilution. ECL chemiluminescence system was used for reaction development (GE- Healthcare, USA).

### Amino acid analyses

Samples adjusted at 1 mg/mL in protein were hydrolyzed with HCl 6 N, during 24 h under vacuum at 110 °C. Amino acid content was assessed by a quantitative amino acid analyzer (Biochrom 30; Biochrom Ltd., Cambridge, UK). The process requires the separation of the amino acids by cation exchange chromatography and derivatization with ninhydrin [18].

### Monosaccharide analyses

Neutral sugars were analyzed by gas chromatography in the form of their alditol acetates [19]. The samples (1 mg) containing polysaccharides were first hydrolyzed with 3 M tri-fluoroacetic acid (121 °C, 1 h). The released monosaccharides were then converted into their corresponding alditol acetates by reduction with NaBH_4_ (Sigma) and subsequent acetylation. Identification and quantification were performed by gas-liquid chromatography on a 6890A instrument (Agilent Technologies, Santa Clara, California, USA) equipped with a flame-ionization detector, using a HP5 fused silica column with He as the carrier gas. Identification was performed on the basis of the coincidence of the retention time of the sample components with those previously measured for known monosaccharide reference standards analyzed under identical conditions and using inositol as internal standard.

### Nuclear magnetic resonance (NMR) studies

NMR spectra were obtained for samples (4 mg/mL in D_2_O) at 298 K. Standard ^1^H NMR and 2D-NMR experiments (TOCSY, HSQC, DOSY) were employed using Bruker Avance 500, 600 and 700 MHz spectrometers (Bruker Ltd., Germany). For heteronuclear 2D-NMR (HSQC), experiments were acquired with 2 K points in a spectral width of 9 ppm in the ^1^H dimension and 110 ppm, center at 55 ppm, in the ^13^C dimension and 256 increments. A relaxation delay of 1 s, and a J-evolution delay corresponding to a J value of 155 Hz were used.^13^C decoupling was achieved by the WALTZ scheme. Standard conditions were employed for the homonuclear 2D-NMR (TOCSY) experiments, with a 50 ms mixing time. The two dimension diffusion ordered spectroscopy (2D-DOSY) experiments were carried out by recording 64–128 scans for each gradient step, a linear gradient of 16 steps between 2 % and 95 %, a diffusion time (big delta) between 0.2 and 0.4 s, and the length of the diffusion encoding gradient pulses (little delta) between 2-4 ms. Dextran markers of different MW were used to perform a calibration curve to obtain the diffusion coefficients (m^2^ s^−1^) as a function of the MW. [20]. All spectra were processed with the protocols implemented in Topspin software (Bruker Ltd). These samples (4 mg/mL in D_2_O) were stored in closed vials at 4 °C during four months to assess the stability of allergen-mannan glycoconjugates (AM) in comparison to native (N) or just polymerized (POL) preparations.

### Specific IgE immunodetection assay

IgE reactivity to the different allergen samples (N, POL and AM) was tested by an immunodetection assay as previously described [21]. Briefly, a total protein amount of 30 μg of the different allergen preparations were placed onto nitrocellulose membranes using a Whatmann Univac vacuum manifold system (GE Healthcare, Canton MA, USA) for allergen binding. The membranes were neutralized with a blocking solution (PBS-0.1 % Tween 20; 3 % defatted milk protein preparation) and incubated (16 h at 4 °C) with individual sera diluted 1/10 from grass pollen allergic patients or with a pool of sera at the same dilution. Afterwards, the membranes were washed with PBS-0.1 % Tween 20 and incubated 1 h at room temperature with anti-human IgE monoclonal antibody diluted 1/5000 followed by a 1 h incubation with a rabbit anti-mouse monoclonal antibody conjugated with peroxidase diluted 1/2000 (Dako, Barcelona, Spain). After washing the membranes with PBS-0.1 % Tween 20, the ECL chemiluminescence system was used for reaction development (GE-Healthcare, Canton MA, USA). Volummograms of the reactive spots were analyzed by scanning densitometry using FUJI FILM MultiGauge v3.0 software.

## Results

### Allergen conjugation with mannan from *S. cerevisae*

**Fig. 1:**
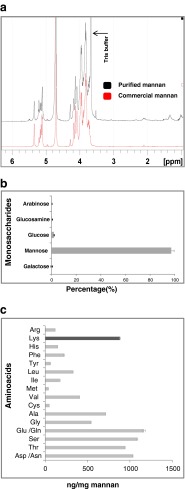
**a** Representation of the different 1 H NMR spectra for the purified mannan and commercial mannan (Sigma M3640). Both the carbohydrate (5–3 ppm) and the aliphatic protein regions (3-0 ppm) are displayed; **b** Monosaccharide analysis in the purified mannan (%); c) Amino acids analysis representation in the purified mannan (ng/mg), the black column showing lysine. Results for b and **c** are expressed as the mean of duplicates ± SD.

Conjugation of allergens to the purified yeast mannan was performed with glutaraldehyde in a single step as decribed in Material and Methods. The purified mannan was very similar to that commercially available as detected by NMR (Fig. 1a), contained almost only mannose (97 %) (Fig. 1b), and contained lysine (883 ng/mg mannan) within other aminoacids (Fig. 1c). For conjugation, different allergen:mannan ratios were used (see Material and Methods). By measuring total carbohydrates, it was found that the AM fraction contained up to 60 % carbohydrates. The lowest amount of mannan to achieve the highest carbohydrate content was obtained at 1:0.5 (allergen:mannan) ratio. As higher amounts of mannan (1:1 and 1:4 ratios) did not increase that percentage, the 1:0.5 ratio was established for further studies.

### Analytical studies of the AM fraction

**Fig. 2:**
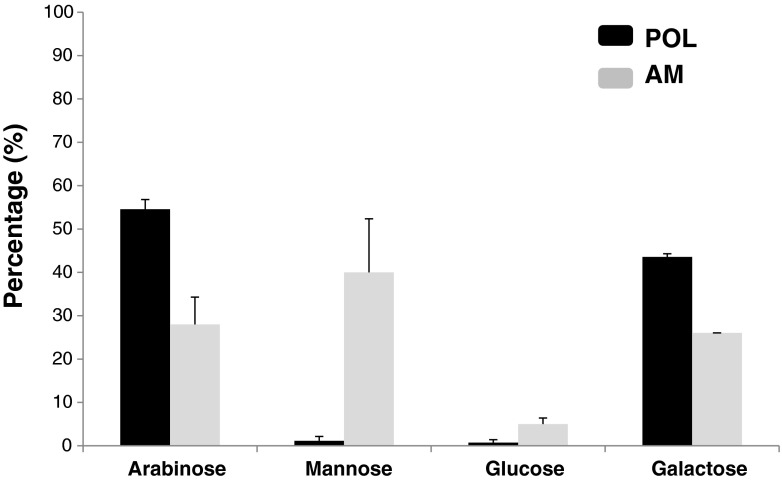
The percentage of monosaccharides in fraction AM (allergen-treated with glutaraldehyde in the presence of mannan) and fraction POL (allergen-treated with glutaraldehyde in the absence of mannan). POL and AM were fractions recovered in the retentate after tangential ultrafiltration (100 kDa pore size membranes). Results are expressed as the mean of duplicates ± SD

Figure 2 shows the percentage of neutral monosaccharides in the AM fraction in comparison with the same fraction (POL) obtained by treating allergens with glutaraldehyde without mannan. As could be expected a strong increase (over 20-fold) of mannoses was seen in the AM fraction, rising to up 40 % of relative content, whereas the other main sugars (arabinose and galactose) decreased. Some glucose was also observed probably due to variability of the crude extracts where its presence has been described [22].

**Fig. 3:**
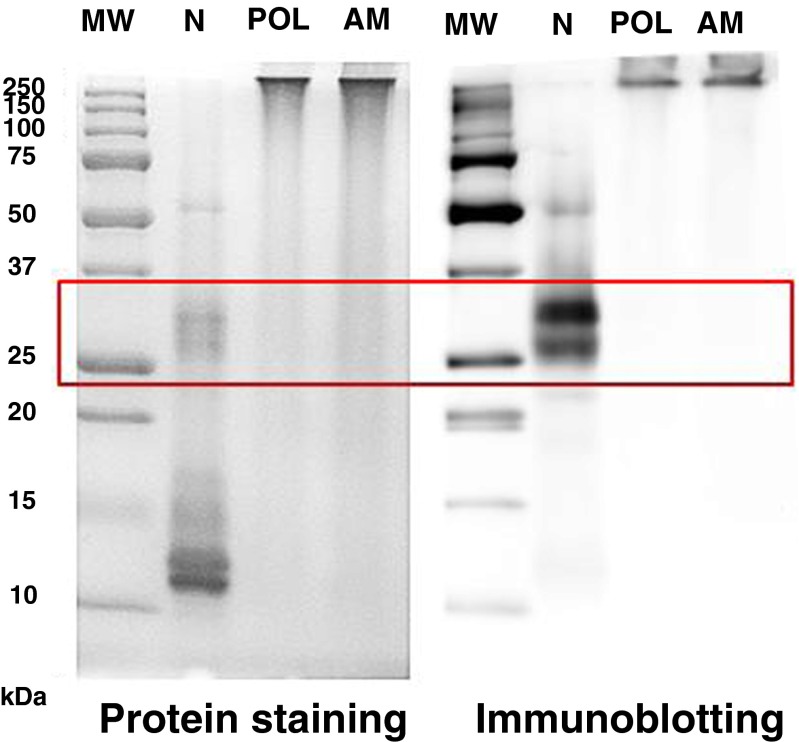
Protein (protein staining: Coomassie blue) and allergen (immunoblotting: IgE) patterns of *Phleum pratense* allergen preparations as detected in SDS-PAGE and Western blot. Lane N: native allergen extract; Lane POL: glutaraldehyde-treated allergen without mannan; Lane AM: glutaraldehyde-treated allergen with mannan. AM and POL were fractions recovered in the retentate after tangential ultrafiltration (100 kDa pore size membranes)

Figure 3 shows the protein profile of AM fraction by gel electrophoresis as detected by protein staining (Coomassie blue) and immunoblotting. Similar to the POL fraction, no bands were detectable in any assay within the gel, in contrast to the lanes loaded with the native allergen (N), in which major allergens from *Phleum pratense* (red box) were readily seen. The results indicated that molecules in the AM fraction were extensively polymerized as did occur when allergens were treated with glutaraldehyde in the absence of mannan.

### Structural studies of the AM fraction

**Fig. 4:**
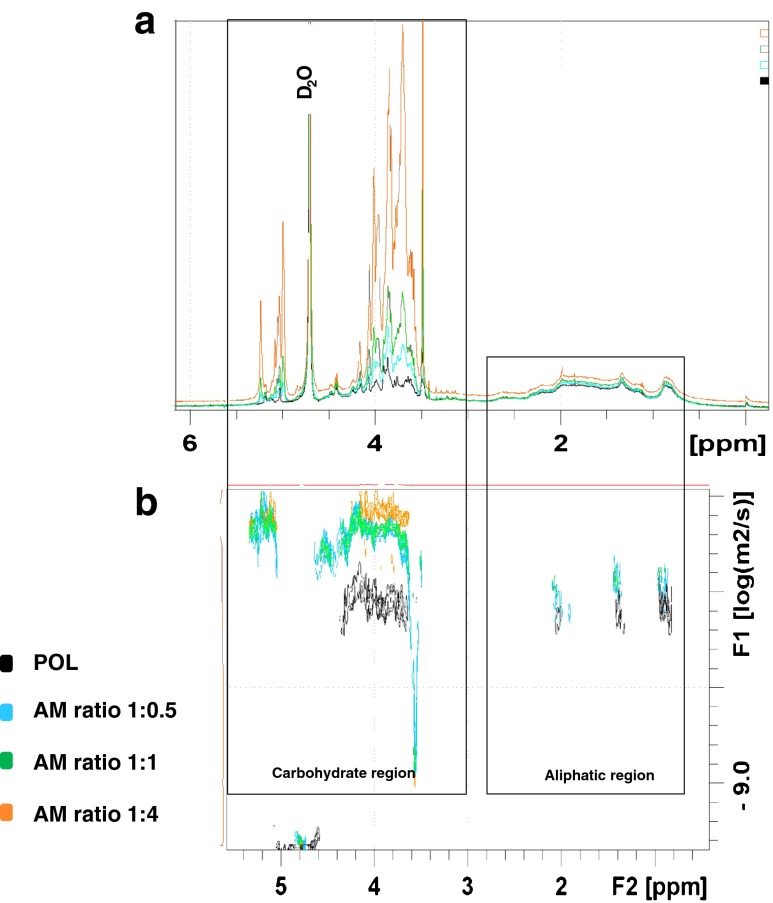
NMR analysis for the different *Phleum pratense* allergen preparations. **a** Superimposition of the different ^1^H NMR spectra recorded. Both the carbohydrate (5–3 ppm) and the aliphatic protein regions (3-0 ppm) are displayed; **b** Superimposition of the different DOSY experiments recorded. Carbohydrate (5–3 ppm) region are displayed. AM (allergen-treated with glutaraldehyde in the presence of mannan); POL (allergen-treated with glutaraldehyde in the absence of mannan). AM and POL were fractions recovered in the retentate after tangential ultrafiltration (100 kDa pore size membranes).

The structure of AM fraction was addressed by NMR as described in Material and Methods. As shown in Fig. 4a, the saccharide signals in the NMR spectra were increased in fraction AM (different conjugation ratios) as compared with the control fraction (POL). 2D-DOSY was performed to detect the presence of heterogeneity, as well as to estimate the average size of the different components in the allergen-mannan conjugates (Fig. 4b). As shown, the different AM fractions showed larger sizes than the POL fraction without mannan, pointing to supramolecular entities formed by association between the protein allergens and mannan.

**Fig. 5:**
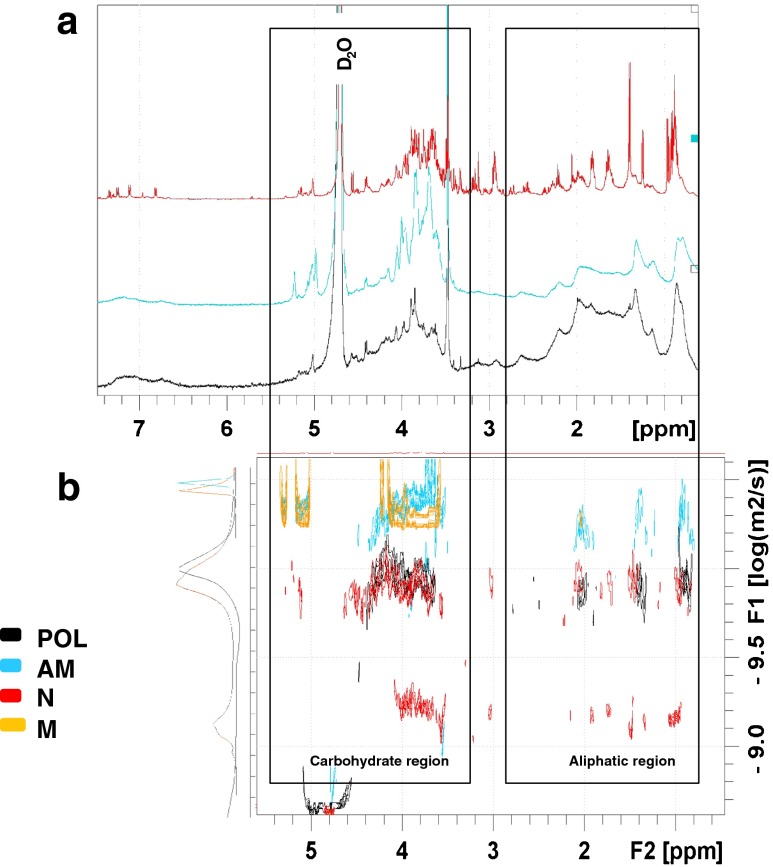
NMR analysis for the different *Phleum pratense* allergen preparations (N, POL, AM) and purified mannan (M). **a** Superimposition of the different ^1^H NMR spectra recorded for the different samples. Both the carbohydrate (5–3 ppm) and the aliphatic protein regions (3-0 ppm) are displayed; **b** Superimposition of the different ^1^H NMR spectra and DOSY experiments recorded for the different samples. Both the carbohydrate (5–3 ppm) and the aliphatic protein region (3-0 ppm) are displayed. Monodimensional DOSY projections represented in the “Y” axis. AM (allergen-treated with glutaraldehyde in the presence of mannan); POL (allergen-treated with glutaraldehyde in the absence of mannan); N (native allergen extract). AM and POL were fractions recovered in the retentate after tangential ultrafiltration (100 kDa pore size membranes).

Figure 5a shows that NMR signals for the different spectral regions showed a constant and significant broadening in the glutaraldehyde-treated samples (AM and POL fractions) as compared with the untreated allergen (N), suggesting the existence of molecular heterogeneity and/or fast transverse relaxation, which is associated with large molecular size entities. The analysis of the translational diffusion 2D-DOSY spectrum further demonstrated the increase of the average molecular size in the glutaraldehyde-treated samples, remarkably in the AM fraction. In this particular case, even larger molecular entities could be inferred from the 2D-DOSY spectra (Fig. 5b). To assess more accurately these size differences, a semi-quantitative molecular weight estimation was carried out in DOSY experiments using the self-diffusion coefficient D (m^2^ s^−1^) and uncharged saccharides (dextran) as standards [20, 23]. The AM fraction showed the highest molecular weight, approximately 5-fold above the POL fraction and 25-fold above the average of the untreated allergen (data not shown).

**Fig. 6:**
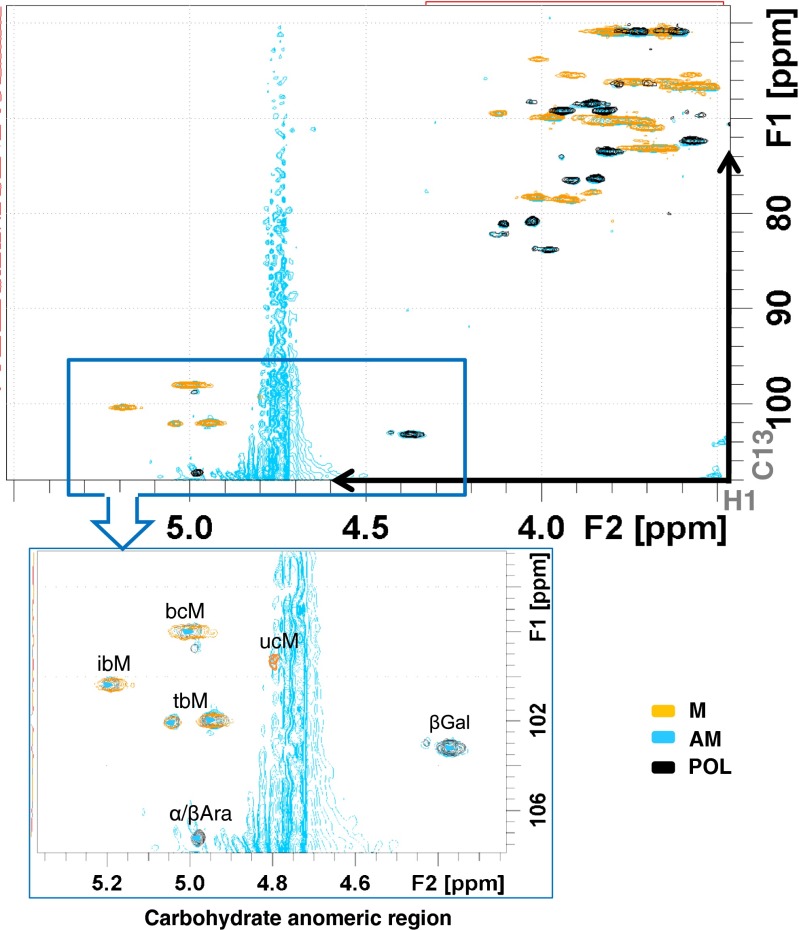
NMR analysis for the different *Phleum pratense* allergen preparations: allergen-treated with glutaraldehyde in the presence of mannan (AM); allergen-treated with glutaraldehyde in the absence of mannan (POL) and purified mannan (M). Superimposition of the different ^1^H-13C HSQC spectra: AM (blue); POL (black) and M (yellow). Signals from Arabino-galactan following assignment by Brecker et al*.* [25]; Signals from Mannans following assignment by Vinogradov et al*.* [24]. Assignment of the carbohydrate anomeric zone: α/βAra (α/β arabinose), βGal (βGalactose), bcM (branched chain mannose), ucM (unsubstituted chain mannose), ibM (2-substituted internal branched mannose), tbM (terminal branched mannose)

Figure 6 shows the analysis of the ^1^H-^13^C HSQC spectra of the allergen-mannan conjugates. As shown, new NMR signals in the carbohydrate region of AM fraction were seen when comparing with the POL fraction in absence of mannan. Some of these are mannan specific signals that have been identified in this spectrum [24], and also arabino-galactan signals described in *Phleum pratense* extracts [25]. This fact unequivocally confirmed the existence of allergen-mannan complexes and therefore the presence of true allergen-mannan glyconjugates in a stable supramolecular structure.

### Stability of allergen-mannan glycoconjugates

**Fig. 7:**
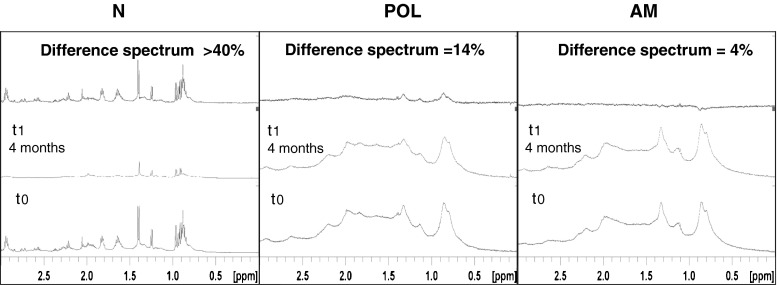
Stability study as detected by NMR analysis for the different *Phleum pratense* allergen preparations stored at 4 °C (N, POL, AM). ^1^H NMR spectra was recorded immediately after their preparations (t0) and four months later (t1). The intensity differences of the key NMR signals between t0 and t1 are highlighted as a percentage (upper part). AM (allergen-treated with glutaraldehyde in the presence of mannan); POL (allergen-treated with glutaraldehyde in the absence of mannan); N (native allergen extract). AM and POL were fractions recovered in the retentate after tangential ultrafiltration (100 kDa pore size membranes)

To assess the stability of the allergen-mannan glycoconjugates contained in AM fraction, its structure was analyzed by NMR just after the conjugation process (t0) and 4 months later (t1). Samples in solution with heavy water (D_2_O) were stored at 4 °C. These included also samples of untreated native allergen (N) and glutaraldehyde-treated allergen without mannan (POL) for comparison purposes at the same protein concentration and storage conditions. As shown in Fig. 7, the difference of NMR signals in the AM fraction conjugated with mannan decreased by only 4 % at t1. By contrast, at that time, a remarkable signal loss was scored for the native allergen (N) (above 40 %) and 14 % for the POL fraction (polymerized allergens without mannan). These results indicated that the structure of allergen-mannan glycoconjugates obtained in fraction AM is remarkably stable as compared with the native allergen, but also more stable than the polymers obtained by treating the allergens with glutaraldehyde without mannan.

### IgE reactivity with allergen-mannan glycoconjugates

**Fig. 8:**
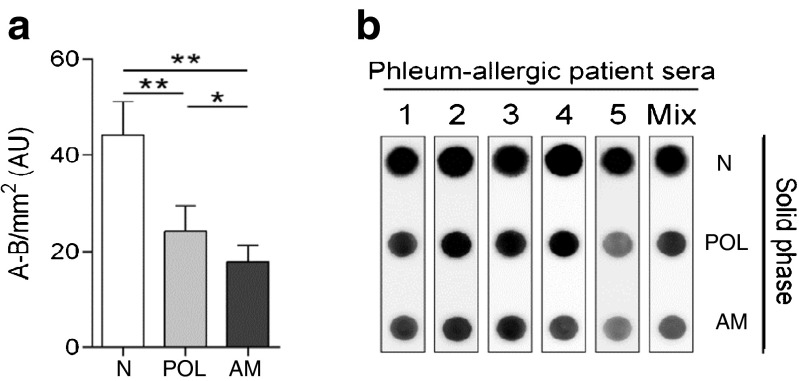
IgE reactivity with the different *Phleum pratense* allergen preparations (N, POL, AM) as measured by dot blot. **a** Quantification of the IgE binding from sera of grass pollen allergic patients by scanning (mean values ± SD); **b** Dot blot results obtained for 5 individual sera and a pooled serum from patients allergic to grass pollen. N (native allergen extract); POL (allergen-treated with glutaraldehyde in the absence of mannan); AM (allergen-treated with glutaraldehyde in the presence of mannan). AM and POL were fractions recovered in the retentate after tangential ultrafiltration (100 kDa pore size membranes).

Finally, we were prompted to test if the allergen-mannan glycoconjugates were so hypoallergenic as the conventional allergoids. This was assessed by a quantitative dot blot immunoassay, testing sera from grass pollen allergic patients as described in Material and Methods with the different allergen preparations. As shown in Fig. 8, the IgE reactivity to the allergen-mannan glyconjugate (AM) was significantly less intense than to the native allergen (N), and even less than to the glutaraldehyde-treated allergens without mannan (POL), though in a lower extent. These results indicated that allergen-mannan conjugates were hypoallergenic, and therefore suitable to be used for immunotherapy.

## Discussion

Coupling of allergens to mannan has been suggested as a way to enhance the allergen uptake by DCs and therefore the efficacy of immunotherapy with allergens [10]. This approach could be even more interesting in the case of allergoids, since it has been claimed that glutaraldehyde-modified allergens are less immunogenic than native allergens (non-modified) due to a lower uptake by DCs [11]. The purpose of this study was to obtain glutaraldehyde-modified allergens conjugated to mannan, taking into account that the conventional conjugation procedure using oxidized mannan was not feasible due to the lack of free amino groups in the allergoids [13].

Here we show that stable allergoids conjugated with mannan can be produced by treating with glutaraldehyde a mixture of allergen and non-oxidized mannan. These glycoconjugates are likely formed through the reaction of the di-aldehyde with the lysine free amino groups of proteins derived from both the allergen and the mannoprotein, i.e., the protein tail linked to the mannan carbohydrate backbone [26]; thus, bridging both molecules by means of glutaryl-diimine bonds. The presence of detectable lysine in our purified mannan preparation (almost 1 μg per mg of mannan) supports such a mechanism. Of note, lysine was also present in all commercially available mannan from *Saccharomyces cerevisae* tested so far (data not shown). The structural studies by NMR show a clear interaction between the allergen and mannan after glutaraldehyde treatment, resulting in high molecular weight complexes. In fact, the broadening of the signals in the allergen-mannan samples indicates a size increase of the complexes as also noted by SDS-PAGE. This is corroborated in translational diffusion DOSY NMR spectra. These complexes showed the lowest diffusion coefficient, as compared with untreated native allergen or glutaraldehyde-treated allergen without mannan, indicating the presence of a molecular entity of larger size. This also evidenced a stable interaction between the allergoid and mannan, since both parts of the spectrum (the carbohydrate and protein areas) show the same diffusion coefficient. Additionally, NMR studies based on HSQC (heteronuclear correlation ^13^C-^1^H) experiments confirmed such interaction, since the allergen-mannan conjugates had the characteristic carbohydrate signals of mannan and also of the intrinsic carbohydrates of the allergen. It should be noted that, in HSQC experiments, every C-H pair of the molecular entity displays a signal with well-defined position, depending on its chemical environment. Thus, the complete set of signals compose a molecular fingerprint, which can be ascribed to a given component [27]. Interestingly, the allergen-mannan glycoconjugates prepared with glutaraldehyde seem to be fairly stable during at least several months, as deduced by their subtractive spectrum by NMR. In fact, they were more stable than the untreated native allergen or the glutaraldehyde-treated allergen without mannan. The remarkable structural stability of allergen-mannan glycoconjugates is clearly an advantage from the perspective of a vaccine development, because the possibility of a longer shelf life.

We have also shown that the allergen-mannan glycoconjugates prepared with glutaraldehyde display a reduced allergenicity. The extent of reactivity of these glycoconjugates with specific IgE was even lower than that observed for the conventional allergoids, i.e., polymerized allergens without mannan. The higher molecular weight of allergen-mannan conjugates may explain this fact, since it is considered that the reduced IgE reactivity with the allergoids depends on the loss of accessibility (steric hindrance) of the antibodies to the allergen epitopes after polymerization [28].

It is important to note that the conjugation approach we describe here preserves the carbohydrate structure of mannan without denaturation, in contrast to the widely used oxidative methods [29]. Oxidation breaks the mannopyranose rings within the polymannose backbone producing remarkable structural consequences [30–32], that may affect its biological properties such as the interaction with antibodies and lectins [33–35]. This is of utmost importance when considering the interaction of mannoses with their corresponding lectin receptors on DCs. In fact it has been already shown that the state of oxidation of mannan may modify the behavior of these cells upon activation [36]. Functional studies from ourselves point to the same direction (Sirvent *et al.*, submitted for publication) supporting the suitability of the conjugation approach we describe here to produce neoglycoconjugates with mannan to be used in the development of novel vaccines.
